# Validation of the Lifetime Incidence of Traumatic Events (LITE-S/P) Questionnaires in Children and Adolescents in Slovenia

**DOI:** 10.3389/fpsyt.2021.665315

**Published:** 2021-07-01

**Authors:** Katarina Uršič, Valentin Bucik, Simona Klemenčič, Nataša Bratina, Tadej Battelino, Klemen Dovč, Maja Drobnič Radobuljac

**Affiliations:** ^1^Department for Haematology and Oncology, University Children's Hospital, University Medical Centre Ljubljana, Ljubljana, Slovenia; ^2^Department of Psychology, Faculty of Arts, University of Ljubljana, Ljubljana, Slovenia; ^3^Department for Endocrinology, Diabetes and Metabolic Disease, University Children's Hospital, University Medical Centre Ljubljana, Ljubljana, Slovenia; ^4^Faculty of Medicine, University of Ljubljana, Ljubljana, Slovenia; ^5^Unit for Intensive Child and Adolescent Psychiatry, Department for Mental Health, University Psychiatric Hospital Ljubljana, Ljubljana, Slovenia

**Keywords:** traumatic events, lifetime incidence of traumatic events, scale translation, scale validation, children

## Abstract

**Introduction:** A traumatic event is an extremely threatening and frightening experience in an individual's life. Children who are exposed to traumatic events are twice as likely to develop a mental disorder. Screening can provide insight into the traumatic experience of children, identifying those eligible for further evaluation, and support. With this aim, we evaluated the psychometric properties of the Lifetime Incidence of Traumatic Events questionnaire (LITE) in Slovene by calculating retest reliabilty, construct validity (cross-informant agreement) and external validity, where we calculated the correlation of the number of differenet traumatic events with psychopathological symptoms.

**Methods:** 280 child-parent pairs (children aged 11.3 ± 2.2 years) from various Slovenian primary schools participated in the study. They were divided into two groups: 180 healthy primary school students and 100 children with Type 1 Diabetes (our study was a part of a larger study *The Influence of Psychobiological Adversity on Children and Adolescents with Type 1 Diabetes Study*). Two versions of the LITE questionnaire were used. Children completed the child report (LITE-S) and parents the parent report (LITE-P) version. After 4 weeks, 117 children, and 114 parents filled out the LITEs again. External validity was assessed using the Youth Self Report and Child Behaviour Checklist syndrome-oriented scales.

**Results:** Retest reliability for individual scales was *r* = 0.469–0.639 (ρ = 0.443–0.636; *p* < 0.001), but higher for individual items (κ = 0.263–0.821; *p* < 0.001). Correlations between reports from parents and children were *r* = 0.313–0.345 (ρ = 0.317–0.348; *p* < 0.001). The number of different events experienced by children correlated significantly with the measured depressive—anxiety, and posttraumatic stress disorder symptoms.

**Conclusions:** Based on our results, the LITE-S and LITE-P “All events” scale have acceptable psychometric properties for use in research and in clinical practise screening. We recommend looking at single items, taking into consideration the responses from both the child and the parent for more precise information. To improve the precision of the psychodiagnostic capacity of the questionnaire, further research on various populations should be performed.

## Introduction

Traumatic events are extremely threatening and frightening experiences in an individual's life that surpass regular stressful events in intensity or nature (1). The nature of the event and extent of the trauma do not necessarily correlate (2, 3).

Two-thirds of children experience one or more traumatic events by the age of 16 (1, 4). A child can experience an event as highly traumatic and react severely to it, even though adults may not perceive it as such (5). Conversely, a child may not experience an event as traumatic, if he or she does not perceive it as dangerous to him/herself or others. Children can experience and react to the same event differently, depending on their understanding of the event, on its nature and intensity, on the importance of the event for the child, as well as on their developmental stage, resilience, the cultural and social factors in their environment, their support network, previous traumatic experience and family issues (3).

Children who are exposed to traumatic events are twice as likely to develop a mental disorder later in life (4). Accumulation of traumatic events reduces the capacity for recovery and stress management, and increases the likelihood of severe and long-lasting consequences (1).

Shortly after a traumatic event the majority of children display behavioural changes or distress (3). In the case of an isolated event, they usually return to their previous level of functioning after a few weeks or months, develop resilience and continue to develop normally (3, 5, 6). However, a minority of children and adolescents develop serious psychological symptoms, which interfere with their everyday functioning, such as posttraumatic stress disorder (3). Some may suffer from higher levels of depression, anxiety, behavioural problems, and other disorders (4). Researchers have found a positive correlation between the number of traumatic events experienced and symptoms of anxiety, depression, posttraumatic stress disorder, externalising problems and the risk of the development of mental disorders (7, 8). The age at occurence and the child's perception of the event also play an important role in the development of mental health problems (5).

Research also shows favourable outcomes in childhood trauma survivors called posttraumatic growth, which occurs due to the effort invested into recovery (9). Posttraumatic growth results from changes in self-perception, where a person sees his or herself as someone who successfully overcame the traumatic event (10).

It is important that individuals exposed to traumatic events (especially children) receive early interventions aimed at successfully overcoming the experienced trauma, instead of risking a deterioration in mental health (11). Regular screenings should be performed to determine which children have been exposed to trauma. A simple screening instrument, which includes various traumatic childhood events, can help us gain insight into traumatic experiences in youth (1).

The Lifetime Incidence of Traumatic Events questionnaire (LITE) is a short checklist for screening and assessing the exposure to trauma in children and adolescents (12). It covers a broad range of potentially upsetting situations that can cause trauma to children and adolescents, such as a car accident, fire, death of a family member, exposure to threats, sexual assault, or witnessing violence.

It successfully predicted the degree of posttraumatic stress disorder in the Child Report of Posttraumatic Symptoms and Parent Report of Posttraumatic Symptoms (12). In summary, the LITE assesses the number of the traumatic events, their impact on the child and successfully predicts the degree of posttraumatic stress disorder on the Child Report of Posttraumatic Symptoms and Parent Report of Posttraumatic Symptoms. However, its psychometric properties haven't been thoroughly researched yet and are described in **Materials and Methods**. There are a few other instruments used for the assessment of exposure to potentially traumatic events with similar psychometric properties (13–16). The advantages of the LITE compared to other available measures are its brevity, that it can be used as a paper-and-pencil measure, that it covers the main potential traumatic events and that it has a wide age range. It also covers the age of the event's first occurrence and the child's perception of the impact of the specific event, which are, according to research, important for discovering children at risk (5).

The aim of the present study was to translate and validate the LITE for the use with children and adolescents in Slovenia.

## Materials and Methods

### Study Design, Study Population and Procedures

This cross-sectional study was a part of a larger study *The Influence of Psychobiological Adversity on Children and Adolescents with Type 1 Diabetes Study (IPA1D Study)* conducted at the Department for Endocrinology, Diabetes and Metabolic Disorders, University Children's Hospital Ljubljana. The protocol of the study was approved by the National Medical Ethics Committee of the Republic of Slovenia (No. 60/08/13) and reported in part in the US NLM ClinicalTrials.gov Trial Nr.: NCT02575001.

Of the participants, 180 were healthy primary school students from various Slovene primary schools (13.3%participation rate), and 100 were children with type 1 diabetes attending primary school (81.9 % participation rate). There were 150 children and adolescents (100 with Type 1 Diabetes and 50 healthy children) in the initial IPA1D Study. Subsequently we added 130 primary school students to obtain retest measures for the LITE.

The families of the healthy children were invited to the study by school counsellors or the study researchers by handing out written materials and explaining the study at the regular school meetings for parents and children upon approval by the school headmasters/mistresses. The families of children with type 1 diabetes were invited by one of their diabetologists; those who agreed to participate were assessed at one of their regular visits to the diabetes clinic.

The main inclusion criterion was being 8–15 years old, while 5–15 years is the age of children enrolled in primary school in Slovenia. The main exclusion criteria were intellectual disability or active psychosis. Participation was voluntary and anonymous. Once the participants and their parents agreed to participate, they signed informed consents/assents, and completed the questionnaires. For the child-parent pairs who agreed to participate in the re-evaluation, the date of re-evaluation was agreed upon in advance and they copied and saved the research numbers of their questionnaires. They filled out the second LITE evaluations under the same study numbers.

The participants were given two sets of questionnaires. In the first group, the children in the IPA1D Study (100 children with type 1 diabetes and 50 healthy children) completed the LITE–S and the Youth Self Report (YSR). Their parents completed the general questionnaires, the LITE–P and the Child Behaviour Cheque List for Ages 6–18 (CBCL/6-18). In the second group, recruited for the purpose of measuring the retest reliability, 130 healthy children completed the LITE–S and their parents completed the general questionnaires and the LITE–P in the first evaluation. This group was given the LITE–S and the LITE–P for a second evaluation after 4 weeks (117 children and 114 parents completed the second evaluation).

### Translation Process

We performed a double back translation of the LITE questionnaire with cooperation and final approval of the author of the original version. There were no additional adaptations made to items in our translation as we found them culturally appropriate for children and adolescents in Slovenia.

### Questionnaires

#### General Questionnaire

Parents provided general demographic (sex, date of birth, nationality, etc.) and family information (sibilings, parents' education, and occupation, etc.) on a special demographic questionnaire (17).

#### Lifetime Incidence of Traumatic Events Questionnaire

The questionnaire is available in the self-report (LITE-S), and the parent-report form (LITE-P) (12). It is used to assess potential traumatic events in a child's life for children aged eight or older. It consists of 16 items, where, after acknowledging an event, a person assesses the emotional distress at the time of the event and at the time of the assessment on a 3-point scale (none, some, lots), the number of times the event occured and the age at the event's first occurence. There is also an additional item, where the child can enter one or more additional events not proposed by the questionnaire. Three scales are derived from their responses: (1) the number of reported traumatic events (≫All events≪); (2) the sum of the distress reported at the time of the events (≫Initial Impact≪) and (3) the sum of the distress reported at the time of assessment (≫Lasting Impact≪).

The retest reliability (*r*) for the ≫All events≪ scale in the Swedish population was *r* = 0.76 and for individual items from κ = 0.33 to κ = 0.86 (1, 18). Li et al. (8) extended the questionnaire with four items regarding the loss of a parent due to HIV. They calculated Cronbach alpha for ≫All events≪ (*r* = 0.69), ≫Initial impact≪ (*r* = 0.92) and ≫Lasting impact≪ *(r* = 0.87). In recent years the questionnaire was used in Korean literature, where they report a retest reliability of *r* = 0.80 (*P* < 0.001) (19–21).

#### The Youth Self Report and the Child Behaviour Cheque List for Ages 6–18

The Youth Self Report (YSR) and the Child Behaviour Cheque List for Ages 6–18 (CBCL/6-18) are used to asses emotional and behavioural problems in children and adolescents (22, 23). The former is filled out by adolescents aged 11–18 years themselves and has 112 items, the latter by one of the parents of children or adolescents aged 6–18 years and has 113 items. Responses for both are recorded on a Likert scale: 0 = Not True, 1 = Somewhat or Sometimes True, 2 = Very True or Often True. They consist of the following eight scales: Withdrawn/Depressed, Anxious/Depressed, Somatic Complaints, Social Problems, Thought Problems, Attention Problems, Rule Breaking Behaviour, and Aggressive Behaviour. The scales Withdrawn/Depressed, Somatic complaints and Anxious/Depressed together comprise a broad internalising dimension, whereas Rule Breaking Behaviour and Aggressive Behaviour together constitute an externalising dimension. The Total Problems score can also be derived. Both questionnaires are a part of the Achenbach System of Empirically Based Assessment, which offers a comprehensive assessment of adaptive and maladaptive functioning. Four decades of studies show content, criterion and construct validity and reliability of the questionnaires (22).

### Statistical Analysis

We used descriptive statistics to characterise the sample, as well as a one-way ANOVA and Kruskal-Wallis test for assessing the between-group differences. We compared childrens' responses on LITE-S by diabetes/no diabetes groups; by age groups and by gender. Participants were divided into two age groups according to their developmental stage for those comparisons-−105 children (11 years or less), and 175 adolescents (12 years or more). There were no statistically significant differences between the groups of included children/adolescents with regard to the general or demographic characteristics or any of the questions on the LITE. There were also, surprisingly, no differences on LITE-S between diabetes/no diabetes groups. Therefore, the group was considered homogenous and comparable in the following analyses. To evaluate the retest reliability, cross-informant agreement and external validity we used Spearman's and Pearson‘s coefficients of correlation as not all distributions were normal. We calculated Cohens Kappa (κ) to assess retest reliability and cross-informant agreement of individual items. To clarify cross-informant agreement, we also performed a discriminant analysis, which helped us understand which items have the strongest distinguishing power for our two groups of reporters (children and parents). The level of significance was set at 0.01. Statistical analyses were performed using the Statistical Package for the Social Sciences (SPSS) for Mac OS software (Version 22.0. Armonk, NY, USA: IBM Corp).

## Results

### Study Participants' Characteristics

The descriptive statistics used on our sample are summarised in Tables 1, 2. The frequency of reports for individual items on the questionnaire reported by parents and children are presented in Table 3. The most frequent event reported by children and parents is “Someone in the family in the hospital (hurt or sick).”

**Table 1:** Descriptive statistics for the sample and traumatic events scales reported by the children and the parents (*N* = 280).

	***N* (%)**	**Mean ± SD**	**Min–Max**	**Skewness ± SE**	**Kurtosis ± SE**
Male	130 (46.43)	–	–	–	–
Female	150 (53.57)	–	–	–	–
Diabetes	100 (35.71)	–	–	–	–
Healthy	180 (64.29)	–	–	–	–
Grade 3	59 (21.1)	–	–	–	–
Grade 4	36 (12.9)	–	–	–	–
Grade 5	61 (21.8)	–	–	–	–
Grade 6	28 (10.0)	–	–	–	–
Grade 7	32 (11.4)	–	–	–	–
Grade 8	24 (8.6)	–	–	–	–
Grade 9	31 (11.1)	–	–	–	–
1st year high school	9 (3.2)	–	–	–	–
LITE-S—all events	–	3.42 ± 1.83	0–10	0.50 ± 0.15	0.38, 0.15
LITE-S—initial impact	–	4.37 ± 2.90	0–13	0.58 ± 0.15	−0.09, 0.15
LITE-S—lasting impact	–	1.86 ± 1.91	0–10	1.38 ± 0.15	2.34, 0.15
LITE-P—all events	–	2.41 ± 1.69	0–8	0.66 ± 0.29	0.32, 0.29
LITE-P—initial impact	–	2.90 ± 2.52	0–12	1.00 ± 0.29	0.73, 0.29
LITE-P—lasting impact	–	0.95 ± 1.40	5–7	1.62 ± 0.29	2.10, 0.29

**Table 2:** Detailed descriptive statistics on gender, grade and diabetes.

	**Diabetes *N* (%)**	**Healthy *N* (%)**	**Girls *N* (%)**	**Boys *N* (%)**
Age (Mean ± SD)	12.25 ± 2.13	10.70 ± 1.95	11.38 ± 2.30	11.09 ± 1.98
Grade 3	12 (4.3)	47 (17.7)	32 (11.4)	27 (9.6)
Grade 4	7 (2.5)	29 (10.4)	19 (6.8)	17 (6.1)
Grade 5	17 (6.1)	44 (15.7)	23 (8.2)	38 (13.6)
Grade 6	12 (4.3)	16 (5.7)	18 (6.4)	10 (3.6)
Grade 7	13 (4.6)	12 (6.8)	16 (5.7)	16 (5.7)
Grade 8	18 (6.4)	6 (2.2)	15 (5.4)	9 (3.2)
Grade 9	12 (4.3)	19 (6.8)	22 (7.9)	9 (3.2)
1st year high school	9 (3.2)	–	5 (1.8)	4 (1.4)

**Table 3:** Number of children and parents reporting an event and frequency of individual traumatic events reported by children and parents (*N* = 280).

	**LITE-S**		**LITE-P**	
	***N***	**%**	***N***	**%**
Been in a car accident	42	15.00	40	14.29
Been hurt in another kind of accident or sick in a hospital	145	51.79	112	40.00
Seen someone else get hurt	153	54.64	54	19.29
Someone in the family in the hospital (hurt or sick)	181	64.64	154	55.00
Someone in the family died	127	45.36	124	44.29
Friend very sick, hurt or died	52	18.57	13	4.64
Been in a fire	5	1.79	4	1.43
Been in hurricane, tornado or mudslide	40	14.29	25	8.93
Parents broke things or hurt each other	12	4.29	16	5.71
Parents separated or divorced	38	13.57	42	15.00
Been taken away from family	1	0.36	1	0.36
Been hit, whipped, beaten, or hurt	58	20.71	27	9.64
Been tied up in a small place	6	2.14	2	0.71
Been made to do sex things	2	0.71	–	−
Been threatened	40	14.29	22	7.86
Been robbed (or house robbed)	34	12.14	18	6.43
Other scary or upsetting event	42	15.00	17	6.07

### Retest Reliability

Correlations between the first and second measurements on the LITE-S and the LITE-P scales are summarised in Table 4. We found the strongest correlation for the “All Events” scale, which was in the moderate range. Parental responses were less variable through time. Reports on the impact of events were less reliable; the coefficients showed a fairly wide range.

**Table 4:** Correlations between the first and second LITE assessments (*N* = 114).

	***r***				*****ρ*****			
	**LITE-S**	***p***	**LITE-P**	***p***	**LITE-S**	***p***	**LITE-P**	***p***
All events	0.62	<0.01	0.64	<0.01	0.58	<0.01	0.64	<0.01
Initial impact	0.47	<0.01	0.63	<0.01	0.48	<0.01	0.55	<0.01
Lasting impact	0.57	<0.01	0.56	<0.01	0.44	<0.01	0.60	<0.01

*r, Pearson Correlation Coefficient; ρ, Spearman's Rho Correlation Coefficient*.

Table 5 presents the consistencies in reporting on both measures for the LITE-S and the LITE-P. The children reported most consistently on the items ≫parents separated or divorced≪, ≫been in a car accident≪, ≫been tied up, or locked in a small space≪, ≫parent (or grown-ups) broke things or hurt each other≪, ≫seen someone else get hurt≪, ≫someone in the family died≪, where the κ was moderate. The κ was strong in the parental reports for ≫parents separated or divorced≪. They also reported consistently on items ≫been hit, whipped, beaten, or hurt by someone else≪, ≫someone in the family died≪, ≫been in a car accident≪, ≫been in a hurricane, tornado, flood, or mudslide≪ and ≫been in a hospital≪, where κ was moderate.

**Table 5:** Cohen's Kappa Coefficient (κ) for agreement between the first and second assessment for individual items on both versions of the questionnaire (*N* = 114).

**Items**	**LITE-S**		**LITE-P**	
	**κ**	***p***	**κ**	***p***
Been in a car accident	0.75	<0.01	0.75	<0.01
Been hurt in another kind of accident or sick in a hospital	0.26	<0.01	0.64	<0.01
Seen someone else get hurt	0.64	<0.01	0.37	<0.01
Someone in the family in the hospital (hurt or sick)	0.55	<0.01	0.44	<0.01
Someone in the family died	0.63	<0.01	0.77	<0.01
Friend very sick, hurt or died	0.37	<0.01	0.42	<0.01
Been in a fire	0.56	<0.01	0.49	<0.01
Been in hurricane, tornado or mudslide	0.54	<0.01	0.71	<0.01
Parents broke things or hurt each other	0.66	<0.01	0.56	<0.01
Parents separated or divorced	0.76	<0.01	0.82	<0.01
Been taken away from family	^*^	–	^*^	–
Been hit, whipped, beaten, or hurt	0.42	<0.01	0.79	<0.01
Been tied up in a small place	0.74	<0.01	0.00	<0.01
Been made to do sex things	^*^	–	^*^	–
Been threatened	0.35	<0.01	0.60	<0.01
Been robbed (or house robbed)	0.54	<0.01	0.59	<0.01
Other scary or upsetting event	0.41	<0.01	0.47	<0.01

* *The value was not calculated due to the low number of participant reports*.

On other individual items consistency between the parent and the child reports was weak.

### Internal Consistency

We calculated Cronbach Alpha for all tree scales for both versions of questionnaire, which were expectedly in low range (α = 0.423–0.458).

### Cross-Informant Agreement in Reports

The correlation between the children's and the parents' reports on the LITE scales is presented in Table 6. There was moderate, but statistically significant agreement for all the scales. Table 7 presents the κ for the agreement between the children and the parents on the individual items. We can see the highest agreement between the parent and the child for the items: ≫parents separated or divorced≪, whereas they agree the least on items ≫been in a hospital≪, ≫been threathened≪, ≫other scary or upsetting event≪, ≫seen someone else get hurt≪, ≫been in a fire≪, ≫been hit, whipped, beaten, or hurt by someone else≪, and ≫parent (or grown-ups) broke things or hurt each other≪.

**Table 6:** Correlations between the LITE-S and the LITE-P scales (*N* = 280).

	***r***	***p***	**ρ**	***p***
All events	0.35	<0.01	0.35	<0.01
Initial impact	0.33	<0.01	0.32	<0.01
Lasting impact	0.31	<0.01	0.33	<0.01

*r, Pearson correlation coefficient; ρ, Spearman's Rho Correlation Coefficient*.

**Table 7:** Cohen's Kappa Coefficient (κ) for the agreement between the children and the parents on the individual items (*N* = 280).

	**κ**	***p***
Been in a car accident	0.53	<0.01
Been hurt in another kind of accident or sick in a hospital	0.12	<0.01
Seen someone else get hurt	0.18	<0.01
Someone in the family in the hospital (hurt or sick)	0.40	<0.01
Someone in the family died	0.56	<0.01
Friend very sick, hurt or died	0.06	<0.01
Been in a fire	0.24	<0.01
Been in hurricane, tornado or mudslide	0.40	<0.01
Parents broke things or hurt each other	0.26	<0.01
Parents separated or divorced	0.80	<0.01
Been taken away from family	–	–
Been hit, whipped, beaten, or hurt	0.24	<0.01
Been tied up in a small place	0.0	<0.01
Been made to do sex things	–	–
Been threatened	0.14	<0.01
Been robbed (or house robbed)	0.61	<0.01
Other scary or upsetting event	0.17	<0.01

In Table 8 the results of discriminant analysis are summarised. We performed the discriminant analysis using one discriminant function, which explained 100% of the variance, canonical *R*^2^ = 0.18 (*r* = 0.42). It significantlly differentiated our two groups—parents and children Λ = 0.82, χ2(17) = 101.23, *p* ≤ 0.01. Using our function 68.1% of original grouped cases were correctly classified. We can see that the items that contribute the most to relative differences between child and parent report in one principal discriminant function, the incidence of traumatic events, are ≫seen someone else get hurt≪, ≫friend very sick, hurt or died≪ and ≫other scary or upsetting event≪. The item that contributes the least is ≫parents separated or divorced≪, for which we saw high agreement between the child and parent report.

**Table 8:** Summary of discriminant analysis measures (*N* = 526^*^).

	**Standardised canonical discriminant function coefficient**	**Canonical structure of the discriminant function**	***F***	***p***
Seen someone else get hurt	0.74	0.82	75.68	<0.01
Friend very sick, hurt or died	0.36	0.46	23.58	<0.01
Been hit, whipped, beaten, or hurt	0.16	0.33	12.39	<0.01
Other scary or upsetting event	0.22	0.32	11.82	<0.01
Been hurt in another kind of accident or sick in a hospital	0.08	0.22	5.27	<0.05
Been threatened	0.02	0.21	5.10	<0.05
Been robbed (or house robbed)	0.18	0.21	5.00	<0.05
Someone in the family in the hospital (hurt or sick)	0.03	0.19	4.05	<0.05
Been in hurricane, tornado or mudslide	0.05	0.15	2.40	0.12
Been made to do sex things	0.10	0.13	1.90	0.17
Been tied up in a small place	0.09	0.13	1.82	0.18
Parents separated or divorced	−0.05	−0.08	0.76	0.39
Parents broke things or hurt each other	0.16	−0.07	0.56	0.45
Been in a car accident	0.01	0.03	0.13	0.72
Someone in the family died	−0.07	−0.02	0.04	0.84
Been in a fire	−0.01	−0.01	0.01	0.94
Been taken away from family	−0.02	−0.00	0.00	0.97

* *The responses of both, the children and adolescents as well as the parents were included in the discriminant analysis*.

### External Validity

Table 9 shows the correlations between the LITE scales and the measures of emotional and behavioural problems. There were weak, but statistically significant associations for most of the “All events” scales. The composite scales correlate more highly than the single symptom scales.

**Table 9:** Correlations between the LITE scales and measures of emotional and behavioural problems.

**YSR (*N* = 80)**	**LITE-S**						**LITE-S**					
	**All events**		**Initial impact**		**Lasting impact**		**All events**		**Initial impact**		**Lasting impact**	
	***r***	***p***	***r***	***p***	***r***	***p***	**ρ**	***p***	**ρ**	***p***	**ρ**	***p***
Anxious/depressed	0.30	<0.01	0.32	<0.01	0.35	<0.01	0.28	<0.05	0.32	<0.01	0.23	<0.05
Withdrawn/depressed	0.10	0.40	0.10	0.40	0.18	0.12	0.11	0.33	0.14	0.22	0.16	0.15
Internalising problems	0.24	0.035	0.24	<0.05	0.33	<0.01	0.24	<0.05	0.24	<0.05	0.26	<0.05
Externalising problems	0.30	<0.01	0.23	0.05	0.21	0.06	0.30	<0.01	0.21	0.07	0.17	0.13
Post-traumatic stress problems	0.28	<0.05	0.28	<0.05	0.29	<0.01	0.28	<0.05	0.27	<0.05	0.20	0.8
**CBCL (*****N*** **=** **143)**	**LITE-P**						**LITE-P**					
	**All events**		**Initial impact**		**Lasting impact**		**All events**		**Initial impact**		**Lasting impact**	
	***r***	***p***	***r***	***p***	***r***	***p***	***ρ***	***p***	***ρ***	***p***	***ρ***	***p***
Anxious/depressed	0.26	<0.01	0.28	<0.01	0.28	<0.01	0.27	<0.01	0.25	<0.01	0.26	<0.01
Withdrawn/depressed	0.18	<0.05	0.21	<0.05	0.17	<0.05	0.18	<0.05	0.23	<0.01	0.16	<0.01
Internalising problems	0.30	<0.01	0.30	<0.01	0.31	<0.01	0.30	<0.01	0.29	<0.01	0.26	<0.01
Externalising problems	0.28	<0.01	0.24	<0.01	0.23	<0.01	0.24	<0.01	0.21	<0.05	0.19	<0.05
Post-traumatic stress problems	0.34	<0.01	0.36	<0.01	0.36	<0.01	0.32	<0.01	0.331	<0.01	0.27	<0.01

*YSR, The Youth Self Report Scale (administered to children older than 11 years); CBCL, The Child Behaviour Checklist (administered to parents); r, Pearson correlation coefficient; ρ, Spearman's Rho Correlation Coefficient*.

## Discussion

The present study aimed to translate and validate the LITE questionnaires for use with children and adolescents in Slovenia. The results demonstrated that the LITE-S and the LITE-P instruments have acceptable validity and thus can be used for assessing exposure to traumatic events, and its impact on children and adolescents.

Retest reliability for both versions of the scales was highest for the “All events” scale and somewhat lower for the “Impact” scales. In both versions of the questionnaire, the LITE-S and the LITE-P, the highest retest reliability was for individual items. Both children and parents reported highly consistently on items ≫Parents separated or divorced≪ and ≫Been in a car accident≪. Retest reliability in the children's version was lowered due to reports on items ≫Been hurt in another kind of accident or sick in a hospital≪, ≫Friend very sick, hurt or died≪ and ≫Been threatened≪, whereas in the parents' version it was lowered due to items ≫Seen someone else get hurt≪ and ≫Been tied up in a small place≪. The stability of the sum of different traumas (“All events”) for the LITE-S scale reported by Nilsson and colleagues, as measured by test–retest reliability on 84 subjects after 3 weeks, was found to be *r* = 0.76 and their κ item per item 0.13–0.86 (18). When compared to their study, our retest reliability results were slightly lower for the scale, but in the same range or higher for individual items on the child version. They did not, however, calculate re-test reliability for impact scales or for the LITE-P. We observed that children reported lower results on all the scales on the second measurements, which could be a result of normalisation of events between both measurements and discussing the answers with their parents. The parents on the other hand reported higher results on all the scales on the second measurement, which could be due to getting new information from the children. We could not find any studies reporting on this comparison. Calculated internal consistency was low, which was expected for the type of questionnaire as the distribution is not normal and the items are not expected to be correlated.

Associations between children's and parents' responses on the scales were highest for the “All events” scale and lower in both “Impact” scales. Similar results (low—moderate positive correlations) were found in other studies, when comparing children's, and parents' reports on a self-report questionnaire (24–26). A study by Ceballo et al. (27) showed that the parents perceived the consequences of the traumatic events as less substantial compared to the children and that the inconsistencies in the reports were due to the following: intentional concealment of the information from parents, the parents ignoring the child's concerns, suppression or denial of the events, and because those events became a part of the child's everyday life. They found low, but significant agreement between the child's and the mother's report on three out of 15 post-traumatic stress symptoms (κ = 0.16–0.27). Our results on the “Impact” scales are consistent with these results.

For individual items, agreement was highest for parental divorce, robbery, death in the family and car accident. It was lower for confinement in a small space, illness or death of a friend, accidents and hospital visits, threats, witnessing injury or accident, and other events. Items where the reports of the children and the parents were highly consistent included the events that children and parents experience together, so they are both familiar with the experience. The agreement still was not perfect as the child may be too young to remember certain experiences or the parent didn't find something as significant as the child did. These findings are consistent with the previous research, where Johnson also reported the highest agreement (κ = 0.21–0.48) for events experienced by parents and children together or where the child is forced to report about the event because other institutions (school, police, doctors) are involved (28). On the other hand, there are items with lower agreement, which are also consistent with the study by Ceballo et al., who found rather low agreement on violent events that happened to the child alone. Their κ statistics (<0.30) supported their χ*2* findings that there was generally low agreement between the reports from the mothers and the children on specific incidents of exposure to violence (27). Similar information was found in our study, where items ≫*Been threatened*≪ and ≫*Been hit, whipped, beaten, or hurt*≪, which describe violence in the community, contribute to higher differences between the parent and the child reports.

We found the correlations between the number of the traumatic events experienced and different measures of symptoms of psychiatric disorders to be, as expected, weakly positive and consistent with the previous studies (5, 7, 8, 29).

### Strengths and Limitations

The main strength of this study is the attempt of translation and validation of the instrument measuring childhood trauma exposure for children and adolescents in Slovenia, where there is a known lack of such instruments. A short screening instrument will help Slovene researchers and clinicians recognise children and adolescents at risk for psychological consequences later in life.

The main limitations were a small sample size, the use of different sets of questionnaires on different participants, meaning that the external validity was calculated on a smaller sample, and the fact that a considerable percentage of the participants consisted of patients with type 1 diabetes. Our sample could also be biassed due to voluntary participation in the study, which also resulted in a skewness of our data towards younger participants. Families with traumatised children or with children with psychological problems may have declined participation to prevent exposing their child to another stressor. However, in comparison to the results of the Swedish study (18), a comparable percentage of participants reported on specific traumatic events for most events included in the questionnaire and, in comparison to the results of other similar studies, our participants reported a similar number of traumatic events (19, 30).

## Conclusions

The present study translated and validated a questionnaire designed to screen for traumatic events in a child's life and pave the way for its use in Slovenia. According to our results, the LITE has acceptable reliability for use as a screening instrument in clinical practise and in research. We recommend the use of the “All events” scale and individual items since they showed the most favourable psychometric properties. In order to obtain more precise data on the psychodiagnostic capacity of the questionnaire, further research on various populations should be performed.

Based on the collected data we can divide the traumatic events into two groups: those which most likely happen within the family and those which most likely happen outside the family. For the former, the parent and the child reports are consistent, whereas in the latter they are not as consistent. We have to take into consideration reports from both the parent and the child in order to get the best insight into traumatic events in the child's life. The proposed division could be used in the further research and could potentially strenghten the psychometric power of the questionaire.

## Data Availability Statement

The raw data supporting the conclusions of this article will be made available by the authors, without undue reservation.

## Ethics Statement

The protocol of the study was approved by the National Medical Ethics Committee of the Republic of Slovenia (No. 60/08/13) and reported in the US NLM ClinicalTrials.gov Trial Nr.: NCT02575001. Written informed consent to participate was provided by the participants' legal guardians and informed assents were provided by the participants.

## Author Contributions

KU collected the data and wrote the first draught of the manuscript. VB mentored KU, helped with the analyses and revised the manuscript. SK participated in the design of the study, collection of the data, the analyses, and writing and revision of the manuscript. NB participated in the design of the study, collection of data, and revision of the manuscript. KD participated in the collection of the data and revision of the manuscript. TB participated in the design of the study and the analyses and revision of the manuscript. MDR participated in the design of the study, collection of the data, and writing and revision of the manuscript. All authors contributed to the article and approved the submitted version.

## Conflict of Interest

The authors declare that the research was conducted in the absence of any commercial or financial relationships that could be construed as a potential conflict of interest.
